# A core of differentially methylated CpG loci in gMDSCs isolated from neonatal and adult sources

**DOI:** 10.1186/s13148-022-01247-1

**Published:** 2022-02-21

**Authors:** Isabella Berglund-Brown, Emily Nissen, Devin C. Koestler, Rondi A. Butler, Melissa N. Eliot, James F. Padbury, Lucas A. Salas, Annette M. Molinaro, Brock C. Christensen, John K. Wiencke, Karl T. Kelsey

**Affiliations:** 1 Warren Alpert Medical School, Brown University, Providence, RI USA; 2 Department of Biostatistics and Data Science, University of Kansas Medical Center, Kansas City, KS USA; 3 Departments of Epidemiology, and Pathology and Laboratory Medicine, Brown University, 70 Ship Street, Providence, RI 02912 USA; 4 Department of Epidemiology, Geisel School of Medicine at Dartmouth, Lebanon, NH USA; 5 Departments of Molecular and Systems Biology, and Community and Family Medicine, Geisel School of Medicine at Dartmouth, Lebanon, NH USA; 6 Department of Neurological Surgery, University of California San Francisco, San Francisco, CA USA

**Keywords:** DNA methylation, Immunomethylomics, MDSCs, gMDSCs, Cancer

## Abstract

**Background:**

Myeloid-derived suppressor cells (MDSCs), which include monocytic (mMDSCs) and granulocytic (gMDSCs) cells, are an immunosuppressive, heterogeneous population of cells upregulated in cancer and other pathologic conditions, in addition to normal conditions of stress. The origin of MDSCs is debated, and the regulatory pattern responsible for gMDSC differentiation remains unknown. Since DNA methylation (DNAm) contributes to lineage differentiation, we have investigated whether it contributes to the acquisition of the gMDSC phenotype.

**Results:**

Using the Illumina EPIC array to measure DNAm of gMDSCs and neutrophils from diverse neonatal and adult blood sources, we found 189 differentially methylated CpGs between gMDSCs and neutrophils with a core of ten differentially methylated CpGs that were consistent across both sources of cells. Genes associated with these loci that are involved in immune responses include *VCL, FATS, YAP1, KREMEN2, UBTF*, *MCC-1*, and *EFCC1*. In two cancer patient groups that reflected those used to develop the methylation markers (head and neck squamous cell carcinoma (HNSCC) and glioma), all of the CpG loci were differentially methylated, reaching statistical significance in glioma cases and controls, while one was significantly different in the smaller HNSCC group.

**Conclusions:**

Our findings indicate that gMDSCs have a core of distinct DNAm alterations, informing future research on gMDSC differentiation and function.

**Supplementary Information:**

The online version contains supplementary material available at 10.1186/s13148-022-01247-1.

## Introduction

A heterogeneous population of immune cells, termed myeloid-derived suppressor cells (MDSCs) [1], are defined phenotypically by their immunosuppressive properties. These cells arise due to various normal stressful states and other pathological conditions that induce a condition reminiscent of emergency myelopoiesis [2]. For example, neonates have an under-developed anti-microbial host defense. It is now recognized that MDSCs play an important role in regulating the earliest immune response [3], as they are quite prevalent in cord blood. Similarly, pregnant women have been reported to have expanded populations of gMDSCs, presumably playing a significant role in producing maternal–fetal tolerance [4]. Finally, MDSCs are commonly produced in numerous cancers [5]. In these conditions, the normal myeloid differentiation pathway is altered; the mature pool of myeloid cells becomes depleted, prompting the genesis of MDSCs that are detectable in peripheral blood [6]. MDSCs suppress the proliferation and function of T cells and have impaired migratory properties [7]. In cancers, this is thought to create a favorable microenvironment for tumors to grow while evading the immune system [6].

Since the initial identification of immunosuppressive MDSCs, researchers have focused on further characterizing these cells, including how they are induced in these many stressful states. MDSCs can be divided into monocytic (mMDSCs), and granulocytic (gMDSCs) populations based on surface phenotype [8]. Because of their granulocytic origin, it is commonly thought that gMDSCs harbor an immunosuppressive phenotype that is derivative of the neutrophil lineage [9]. Despite numerous studies to develop specific markers of gMDSCs, there is no consensus immunophenotype. The most common feature used to distinguish these cells is their lower density than granulocytic cells, thus co-purification with peripheral blood mononuclear cells following density gradient centrifugation [10].

The precise mechanisms of differentiation or activation that give rise to MDSCs remain unclear. Since DNA methylation (DNAm) has been explored extensively in studies seeking to define the molecular basis of hematopoietic lineage differentiation [11, 12], as well as in lineage activation (e.g., NK cells) [13], DNAm is an excellent candidate for playing an essential role in generating gMDSCs. Patterns of methylation in promoters, enhancers, and gene bodies can strongly correlate with gene expression [14] and define differentially methylated regions (DMRs) used to predict cell lineage [15, 16]. For example, stable and heritable changes in DNAm, regardless of environmental influences, define lineage in T-cell differentiation [17]. Regulatory T cells have been shown to have stable and invariant methylation marks that define their phenotype [18, 19]. Taken together, these data suggest that DNA methylation analysis could provide powerful markers of differentiation states of polymorphonuclear leukocytes (PMNs) that may help define the nature of gMDSCs.

The few DNAm analyses that have been conducted point to the value of this approach, though prior studies have not explored cells isolated from diverse biologic sources. For example, when comparing methylation of inhibitory/suppressive molecules in MDSCs to antigen-presenting cells, CpG islands in the promoter regions of *TGF-β1*, *TIM-3*, and *ARG1* were highly unmethylated [20]. Here, researchers hypothesize that epigenetic mechanisms could control the genesis of suppressive molecules in the tumor microenvironment that contribute to immune tolerance [20]. In another study looking at infiltrating MDSCs in the context of colorectal cancer, genes associated with DNAm-mediated transcriptional silencing and suppression pathways like WNT were upregulated in these cells [21]. Further, some data suggest that DNAm may play a vital role in the genesis of MDSCs, in addition to function. Researchers have also found that demethylated CpG sites characteristic of dendritic cell differentiation were not demethylated in MDSCs [22]. MDSCs show hypermethylation and gene repression in immunogenic regions important for their immunosuppressive properties, which could specifically be involved in the “switch” from immunogenic to tolerant in a tumor environment [22].

While DNAm of MDSCs has been studied in the context of specific cancer types and immunosuppression, epigenetic characterization of multiple sources of gMDSCs has not yet been done. Given the extreme heterogeneity of neutrophils in different clinical in vivo environments, our central objective was to directly examine the DNA methylome of gMDSCs arising in different biologic contexts to epigenetically phenotype these cells. We followed the recommended phenotypic features of gMDSCs as described in Bronte et al., 2016 to guide the isolation of putative gMDSCs from diverse sources [23]. The approach focused on identifying consistent alterations, allowing us to uncover a core set of DNAm alterations that are associated with the genesis of immune suppression and thus potential markers of this phenotype. To implement this, the DNAm of gMDSCs and neutrophils from two distinct age populations were compared: adult blood, where gMDSCs were collected from cancer patients and a pregnant mother, and from umbilical cord blood. These analyses revealed several robust differences in DNAm, consistent across both the adult and cord blood datasets, where some of the alterations associated with genes that have known immune functions. We then compared the whole blood methylation value for the identified loci in cancer patient studies to test whether they were altered in cancer.

## Materials and methods

### Patient samples (discovery)

**Fig. 1:**
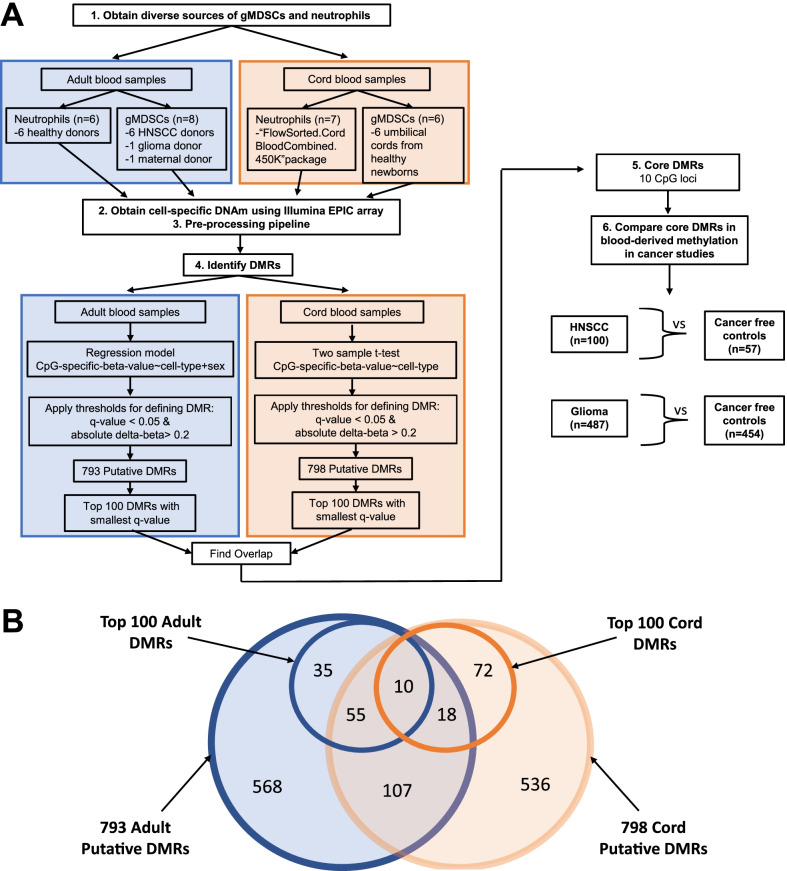
Study design and resulting overlaps of DMRs identified. **A** Diagram of the study design. Briefly, we (1) obtained diverse sources of gMDSCs and neutrophils (2) Measured DNAm with the Illumina EPIC array (3) Pre-processed the datasets (4) Identified DMRs using statistical tests (5) Defined a core set of DMRs and then (6) Tested the core DMRs in DNAm from cancer studies. **B** Venn diagram of putative DMRs identified and the overlaps between the sets. This includes any loci that met the thresholds set and the subset of the top 100 DMRs that were identified by smallest *q*-value. The numbers in the rings are the number of CpG loci in that set

The Institutional Review Boards approved biological sample collections from their respective institutions. Deidentified whole blood samples were obtained from adults following written informed consent; head and neck cancer patient samples were collected from the Dana Farber Cancer Institute (Boston, MA) from newly diagnosed patients before initiation of treatment. Whole blood was obtained from a newly diagnosed glioma patient at the Rhode Island Hospital (Providence, RI), following all applicable IRB guidelines. Cord blood and maternal blood samples were obtained at Women and Infants Hospital (Providence, RI) from discarded cords and consenting pregnant women, following the institutional guidelines. Hospital personnel collected all samples during regular standard of care visits, except for cord blood samples obtained from discarded umbilical cords. Samples were drawn and transported to the laboratory at Brown University within 0.5–3 h. The list of samples included in the analyses is summarized in Fig. 1A.

### Discovery sample processing

Blood was diluted threefold in PBS (pH 7.4, without calcium or magnesium; ThermoFisher cat no 10010023) supplemented with 2 mM EDTA (Fluka cat no 03690-100 ml), and run over a Ficoll density gradient (density 1.077 g/ml; GE Healthcare cat no 17-1440-02). The PBMC fraction, which also contains low-density granulocytic cells, was collected and washed with HBSS (no calcium or magnesium; ThermoFisher cat no 14170112) supplemented with 2 mM EDTA, to remove platelets. The following cell-surface markers were labeled for sorting granulocytes: CD11b-PE (clone REA592; Miltenyi), CD14-eVolve605 (clone 61D3, eBiosciences) or CD14-Superbright600 (also clone 61D3, Thermo), CD15-APC (clone VIMC6, Miltenyi), CD33-PEVIO770 (clone AC104.3E3, Miltenyi), CD66b-FITC (clone REA306, Miltenyi), HLA-DR-PerCPVio700 (clone AC122, Miltenyi), and viability dye efluor450 (eBiosciences). All antibodies (including new batches) were titrated for optimal signal prior to initiation of cell sorting experiments. Gating was optimized using FMO controls before the initiation of sorting experiments. Each sorting session began by setting compensation with fresh compensation controls using beads, either AbC Anti-Mouse Bead Kit (ThermoFisher cat no A10344) or MACS anti-human Igκ Comp Bead kit (Miltenyi cat no 130-104-187). In cases with HNC samples, antibody labeled cells were ‘lightly fixed’ with IC Fixation Buffer (ThermoFisher cat no 00-8222-49) at 4 °C for 10 min, washed, and stored overnight at 4 °C before cell sorting. Cell sorting was carried out at the Brown University Flow Cytometry and Sorting Facility on a 5-laser, 20-parameter BD FACSAria IIIu.

Samples were gated for live, singlet cells, and, based on forward scatter vs. side scatter, the granulocytic fraction containing gMDSCs was gated for CD11b+, CD33+, CD14−, CD15+, HLA-DR- and CD66b+. Neutrophils were collected from 2 ml of whole blood using Magnetic Activated Cell Sorting methods (MACSxpress human neutrophil isolation kit, Mitenyi cat no 130-104-434) with some modification: cells were processed in HBSS (no calcium or magnesium) supplemented with 2 mM EDTA. Neutrophils were negatively selected using two rounds of the kit antibody cocktail, resuspended in supplemented HBSS, checked for viability (trypan blue), and counted. Aliquots of cells were frozen at − 80 °C for later downstream processing and analyses. One aliquot of fresh cells was reserved for antibody labeling and FACS phenotype verification.

### Suppression assay

The suppression assay was carried out for representative gMDSC discovery samples using heterologous gMDSCs and PBMCs (containing target T cells). PBMC aliquots were generated from healthy donor leukoreduction filters obtained from the local blood donation center (Rhode Island Blood Center). White blood cells were back-flushed with 250 ml PBS (no calcium or magnesium) at a rate of approximately 25 ml/min to avoid excessive pressure. The PBMC fraction was collected from a Ficoll gradient as described above (see Sample Processing) and cryopreserved in single experiment aliquots. Just before use, the PBMCs were thawed, washed, labeled with CellTrace Far Red Cell Proliferation Kit (ThermoFisher cat no C34572) *per* manufacturer’s instructions, and stimulated with 30U human IL-2 IS premium grade (Miltenyi cat no 130-097-745) *per* ml of cell suspension and 1ul of Dynabead Human CD3/CD28 T Cell Activation Kit (ThermoFisher cat no 11161D) *per* 1 × 10^5^ (100 ul) PBMCs.

Live gMDSCs were sorted into complete RPMI: RPMI 1640 (ThermoFisher cat no 11875168), supplemented with 10% FBS (ThermoFisher cat no 16000044), 1 × pen/strep/l-glutamine (ThermoFisher cat no 10378016), washed and resuspended at 1 × 10^5^ gMDSCs/100ul medium. Granulocytic MDSCs were co-cultured with activated PBMCs in 96-well tissue culture plates in a 1:1 ratio and cultured under standard conditions (37 °C with 5% CO_2_). After 4–5 days, cells were harvested, activation beads removed, and cells labeled for CD66b to determine gMDSCs, and CD3 (clone BW264/56; Miltenyi), CD4 (clone SK3; BioLegend), and CD8 (clone BW135/80; Miltenyi) to determine T-cell populations. The signal from Far Red dye was used to determine the proliferation of CD4+ and CD8+ cells in the presence or absence of gMDSCs. Proliferation metrics were calculated using the FlowJo 10 Proliferation Tool. Both Division Index (the average number of cell divisions across all cells in the original population, regardless of whether they have divided or not) and the Proliferation index (the number of cell divisions/the number of cells that divided).

### DNA isolation and methylation array of discovery and testing samples

Fresh frozen cell pellets from training samples were extracted for DNA using the Zymo Quick-DNA MiniPrep Plus kit (cat no D4068) per manufacturer’s instructions. To maximize DNA recovery from lightly fixed frozen cells, pellets were subjected to a high heat/high pH method of Campos and Gilbert [24]. Briefly, fixed cells are incubated for 40 min in 0.1 M sodium hydroxide, 1% SDS solution at 99 °C, allowed to cool for 5 min, and organically extracted. Samples were precipitated with sodium acetate (pH 5.s; ThermoFisher cat no R1181) and isopropanol in the presence of GlycoBlue carrier (ThermoFisher cat no AM9516). Testing sample DNA was extracted from whole blood using either Qiagen DNEasy Blood & Tissue Kit (cat no 69504) or Zymo Quick-DNA MiniPrep Plus kit (cat no D4068) per manufacturer’s instructions.

DNA was sent to either the USC Molecular Genomics Core facility or the Avera Institute for Human Genetics to process samples for the Illumina EPIC Human DNA Methylation Beadchip. Both facilities bisulfite convert using Zymo Research products. The DNA from fixed samples was processed by the USC Core and treated with the Illumina FFPE QC and Infinium HD FFPE Restore Solution kits.

The neutrophil adult blood methylation data are from Salas et al. [25], and the neutrophil cord blood methylation data are from publicly available Illumina array data on umbilical blood cells (FlowSorted.CordBloodCombined.450 k) [26].

### Statistical analysis

#### Identifying DMRs

To identify putative DMRs between neutrophils and gMDSCs from adult blood, a series of linear regression models were fit independently to each CpG by modeling CpG-specific methylation beta-values as the dependent variable and cell identity as the independent variable (*n* = 8 gMDSC, *n* = 6 neutrophil) (Fig. 1A). Linear regression models were adjusted for sex. The regression coefficient for cell-type identity and its corresponding *p*-value were recorded. To correct for multiple testing, the resulting *p*-values were adjusted to control the false discovery rate (FDR) using the Benjamini–Hochberg method [24]. As a filtering step, the recommended general-purpose masking from Zhou et al. [27] was used, along with removing CpG loci located on the X and Y chromosomes and removing “ch” and “rs” probes. Putative neutrophil/gMDSC DMRs were defined as the 100 CpG loci with the smallest adjusted *p*-value and an absolute delta-beta (difference in mean beta-value between gMDSCs and neutrophils) greater than 0.2.

Similarly, to identify putative DMRs between neutrophils and gMDSCs from cord blood, a series of two-sample *t*-tests were conducted to test the difference in CpG-specific DNAm between neutrophils and gMDSCs (*n* = 6 gMDSC, *n* = 7 neutrophil) (Fig. 1A). The mean difference between neutrophil methylation and gMDSC methylation was recorded along with the corresponding *p*-value from the *t*-test. Raw *p*-values were adjusted using the Benjamini–Hochberg method [24], followed by filtering using the same process described above, and putative DMRs were defined similarly as for the adult blood. The CpGs in common between these two lists of DMRs from adult and cord bloods, were identified, and these signature core loci formed the basis of subsequent statistical analyses (Fig. 1B).

### Case–control study populations

#### Head and neck squamous cell carcinoma (HNSCC)

We interrogated methylation using the Illumina HumanMethylationEPIC array for whole blood samples from 100 cases diagnosed with incident oropharyngeal squamous cell carcinoma that were randomly selected from a population-based case–control study of head and neck cancer in the greater Boston area that has been described elsewhere [28]. Briefly, 533 incident cases of HNSCC (phase 1, December 1999 to December 2003) and an additional 509 incident cases (phase 2, October 2006 to June 2011) were recruited through major teaching hospitals located in Boston, Massachusetts. Adult cases (> 18 years old) included residents of greater Boston with a confirmed incident diagnosis of HNSCC. Cases were excluded if tumors originated in the lip, salivary gland, nasopharynx, nasal sinus, or cavity. Cases were also excluded if their initial primary tumor was diagnosed more than 6 months prior to study contact. Based on the American Joint Committee on Cancer (AJCC) recommendations, tumors were classified as oral cavity, oropharynx, or larynx. We randomly selected 100 participants with oropharyngeal cancer for the current study (ICD-9 codes 146, 148, 149). Cancer-free controls were frequency-matched to cases on age and gender. Controls were selected by matching their town of residence, age, and sex, as has been previously described [28].

#### Glioma

The case–control San Francisco Adult Glioma Study (AGS) includes 3,164 glioma patients newly diagnosed between 1991 and 2012 who were residents of the SF Bay Area or patients of the UCSF Neuro-oncology clinic and 2,140 subjects without glioma who were residents of the SF Bay Area or patients seen in the UCSF phlebotomy clinic [29]. Controls were frequency-matched to cases by age, race/ethnicity, and gender. Blood samples were collected from glioma patients a median of 100 days after they were histologically diagnosed. For this paper, we included only AGS subjects who had 850 K methylation data available (487 glioma patients and 454 controls).

#### Evaluating the gMDSC signature loci in cancer

To test whether the identified gMDSC core loci were altered in cancer, the whole blood methylation value for these loci were compared in two independent cancer patient studies: HNSCC and glioma (Fig. 1A). For each study, a series of linear regression models were fit independently to each CpG by modeling the CpG-specific whole blood methylation beta-value as the dependent variable and cancer status as the independent variable (*n* = 100 HNSCC cases and *n* = 57 controls; *n* = 487 glioma cases and *n* = 454 controls). The models for comparing case versus control from the HNSCC study also adjusted for cell-type, age, sex, smoking status, drinking status and race. The models for comparing case versus control from the glioma study also adjusted for cell-type, age, sex, smoking status, steroid use, and race. Cell-type was included as the predicted proportions of CD4T cells, CD8T cells, monocytes, neutrophils, and B-cells, obtained via reference-based cell mixture deconvolution [11, 30]. The regression coefficient for cancer status and its corresponding *p*-values was recorded for each model. A CpG was considered to have significantly different whole-blood methylation between cancer cases and cancer-free controls if the *p*-value for cancer status was less than 0.05.

### Statistical analysis software

All analyses were done in R version 4.1.0.

## Results

**Fig. 2:**
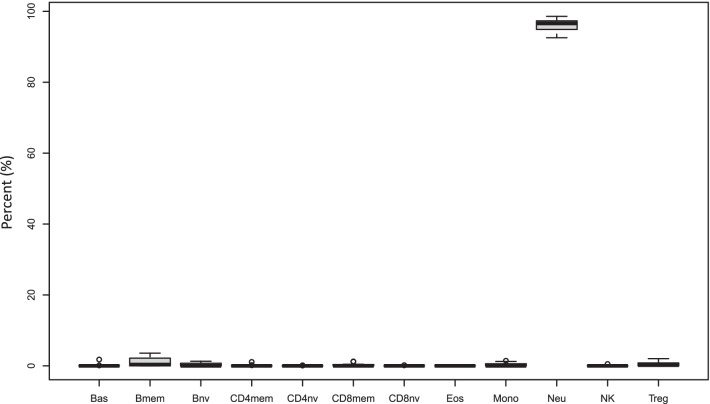
gMDSC sample deconvolution. The relative prevalence of each cell type in the low-density neutrophil fraction. Isolated gMDSCs were arrayed and the resulting data combined and assessed by deconvolution to generate relative proportions for each cell type. The means for each cell type from all donors are shown

The overall study design is shown in Fig. 1A. Briefly, we isolated neutrophils and gMDSCs from diverse sources, profiled their DNA methylation status using the Illumina EPIC array and compared the profiles to discover the changes in methylation that characterized the gMDSCs. After the initial gMDSC isolation we assessed the lineage of cells and Fig. 2 shows the predicted proportions of each of the12 cell-types obtained by reference-based cell mixture deconvolution for each of the gMDSC samples, using the method of Salas et al. [25]. For all isolated gMDSC samples, as expected, the most abundant cell-type was neutrophils (median of 96.6%) (Additional file 1: Table S1). This (presumably) shows that the DMRs used to predict neutrophil lineage are retained in gMDSCs. To ensure our isolation methods captured phenotypically active gMDSCs, representative samples from HNSCC, cord and maternal sources were assessed for their ability to suppress T-cell activity. The suppression assay was carried out using heterologous gMDSCs and PBMCs (containing target T cells). The Proliferation Index (PI) and Division Index (DI) were calculated for CD4 and CD8 T cells in the presence and absence of our isolated gMDSCs from each representative cell source. Both PI and DI were significantly diminished in the presence of gMDSCs, indicating that these cells could suppress normal T cell responses, consistent with the expected gMDSC phenotype (Table 1).

**Table 1:** %Reduction in Division Index (DI) and Proliferation Index (PI) in the presence of gMDSCs from different sources

		CD4		CD8	
PBMC: gMDSC	gMDSC source	% ↓ DI	% ↓ PI	% ↓ DI	% ↓ PI
1:1	Cord blood	58.7	8.6	58.7	11.3
1:1	Maternal	43.6	1.8	10.8	13.6
1:1	HNC	58.0	20.7	63.8	10.3
1:1	HNC	30.2	5.3	58.0	21.4

**Fig. 3:**
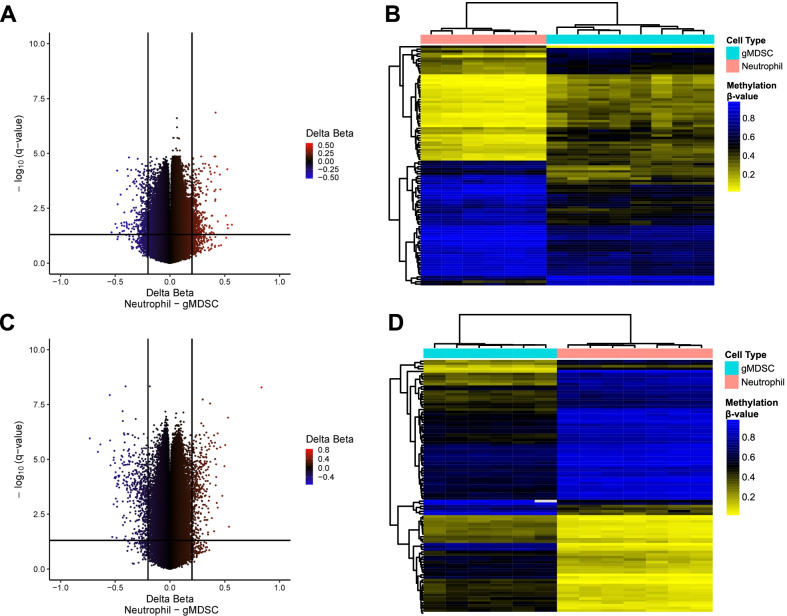
Identifying putative neutrophil/gMDSC DMRs from adult blood samples and cord blood samples. **A** Volcano plot of − log 10(*q*-value) against delta-beta, which represents the difference in mean methylation beta-value between neutrophils and gMDSCs, adjusted for sex, in adult blood samples. The horizontal black bar represents the threshold of significance (*q*-value (FDR) = 0.05) and the two vertical black bars represent the threshold for delta-beta (delta-beta =  ± 0.2). **B** Unsupervised clustering heatmap of the top 100 gMDSC DMRs identified in adult blood samples, defined as the 100 CpG loci with the smallest adjusted *p*-value and absolute adjusted delta-beta greater than 0.2. Each column represents a sample, and each row represents a CpG locus. Above the heatmap, color indicates cell-type (blue = gMDSC and pink = neutrophil). Within the heatmap, color indicates methylation beta-value (blue = *β* of 1, or methylated, and yellow = *β* of 0, or unmethylated). **C** Volcano plot of − log10(*q*-value) against delta-beta, which represents the difference in mean methylation beta-value between neutrophils and gMDSCs in cord blood samples. The horizontal black bar represents the threshold of significance (*q*-value (FDR) = 0.05) and the two vertical black bars represent the threshold for delta-beta (delta-beta =  ± 0.2). **D** Unsupervised clustering heatmap of the top 100 gMDSC DMRs identified in cord blood samples, defined as the 100 CpG loci with the smallest adjusted *p*-value and absolute delta-beta greater than 0.2. Each column represents a sample, and each row represents a CpG locus. Above the heatmap, color indicates cell-type (blue = gMDSC and pink = neutrophil). Within the heatmap, color indicates methylation beta-value (blue = *β* of 1, or methylated, and yellow = *β* of 0, or unmethylated)

Having confirmed the protocols employing density gradients and FACS to isolate gMDSCs with an immunosuppressive phenotype, we sought to identify differentially methylated regions (DMRs) that distinguish between gMDSCs and neutrophils. Initially, both cell subtypes were compared in blood from adult donors, where DNA methylation (DNAm) was measured using the Illumina HumanMethylationEPIC platform. A series of linear models were fit independently to each CpG and used to test differences in DNAm between gMDSCs and neutrophils. The DNA methylation signals were compared at each CpG site and initially sorted based on the magnitude of the difference in methylation beta-values (commonly referred to delta-beta, which ranges from -1 to 1). Figure 3A shows the difference in mean methylation beta-values between neutrophils and gMDSCs, adjusted for sex, for all CpGs in adult blood samples. There were 793 CpGs that met our criteria for differential methylation (*q*-value (FDR) < 0.05 and absolute adjusted delta-beta > 0.2) (Fig. 1B and Additional file 2: Table S2). From these 793 putative DMRs, the top 100 neutrophil/gMDSC DMRs were defined as the 100 CpG loci with the smallest adjusted *p*-value and neutrophil and gMDSC samples were compared using the methylation beta values in an unsupervised clustering heatmap (Additional file 3: Table S3 and Fig. 3B). DNA methylation in leukocytes varies with age, and the age range of the patients who provided the adult blood for gMDSC and neutrophils differed statistically: neutrophil donor mean age was 26.8 years and gMDSC donor mean age, 68.1 years (Additional file 4: Table S4). To mitigate the potential false discovery of gMDSC DMRs attributable to the effects of age and to enhance the sample heterogeneity, we next compared DNAm between gMDSCs and neutrophils in cord blood with the same analytic approach. Figure 3C shows the difference in mean methylation beta-value between neutrophils and gMDSCs from cord blood. There were 798 CpGs that met our criteria of being differentially methylated (*q*-value (FDR) < 0.05 and absolute delta-beta > 0.2) (Fig. 1B and Additional file 5: Table S5). Figure 3D shows a heatmap of the methylation beta-values in gMDSC and neutrophils of the top 100 neutrophil/gMDSC DMRs for each cell type (Additional file 6: Table S6). The presence of non-overlapping putative DMRs illustrates the heterogeneity of these cells. There were 603 non-overlapping putative DMRs identified only in adult blood and 608 non-overlapping putative DMRs identified only in cord blood. (Fig. 1B). The genomic and functional context of these DMRs are also heterogeneous (Additional file 7: Table S7).

**Fig. 4:**
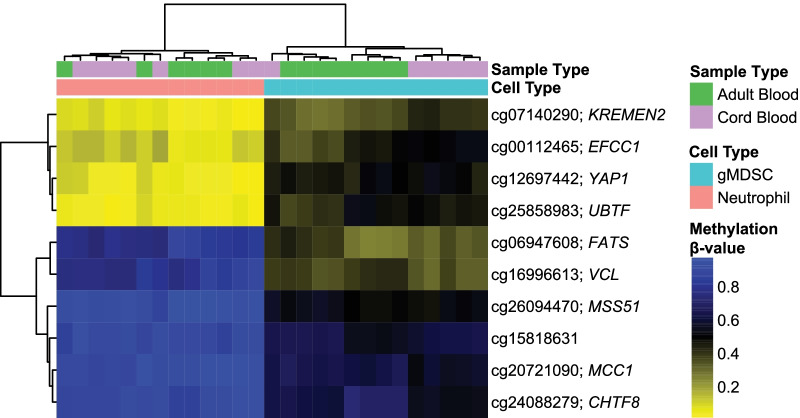
Core of differentially methylated CpG loci between gMDSCs and neutrophils from diverse adult and neonatal sources. Unsupervised clustering heatmap of the core ten gMDSC loci. Each column represents a sample, and each row represents a CpG locus. Above the heatmap, colors indicate sample type (green = adult blood, purple = cord blood) and cell-type (blue = gMDSC and pink = neutrophil). Within the heatmap, color indicates methylation beta-value (blue = *β* of 1, or methylated, and yellow = *β* of 0, or unmethylated)

Despite the great heterogeneity of these cells, there was a core of DNAm alterations specific to gMDSCs. When we compared adult and cord blood gMDSC DMRs, 190 CpGs overlapped (Fig. 1B and Additional file 8: Table S8). Since these 190 DMRs were derived from independent comparisons, we further analyzed these DMRs to assess whether the direction of the change in methylation in the gMDSCs (when compared with neutrophils) was consistent; for 189 of the 190 DMRs, the direction of the change was the same in adult and cord blood. To discover the loci reflecting the most prominent, generalizable differences in DNAm between neutrophils and gMDSC across lifespan, we compared the top 100 DMRs in adult blood with the top 100 DMRs in cord blood and identified an overlap of ten loci (Fig. 1B). These ten loci represent a core of differentially methylated CpG loci across diverse sources. A heatmap generated of these 10 CpGs is shown in Fig. 4. In both adult and cord blood, gMDSCs cluster together, and the neutrophils cluster together across these ten loci. As displayed in Table 2, the directionality of the change in the beta values of these ten loci also is preserved between adult and cord blood. Of the 10 CpG sites in the gMDSC signature loci, nine are associated with specific genes (Table 2).

**Table 2:** 10 CpG overlap between adult blood and cord blood

CpG	Chr	Gene	Genomic context	Delta beta AB	Delta beta CB	*P*-value AB	*P*-value CB	Functional context
cg07140290	chr16	*KREMEN2*	CpG Island	− 0.26	− 0.32	1.91E−08	2.77E−10	TSS200
cg00112465	chr3	*EFCC1*	CpG Island	− 0.30	− 0.33	3.01E−06	3.43E−08	TSS1500
cg12697442	chr11	*YAP1*	CpG Island	− 0.38	− 0.41	2.05E−09	1.35E−09	TSS200
cg25858983	chr17	*UBTF*	North shore	− 0.37	− 0.41	1.24E−07	6.96E−16	5′UTR
cg06947608	chr10	*FATS*	Open sea	0.45	0.41	5.6E−07	1.44E−09	3′UTR
cg16996613	chr10	*VCL*	Open sea	0.39	0.43	6.11E−10	7.00E−09	Body
cg26094470	chr10	*MSS51*	Open sea	0.41	0.38	3.77E−10	1.52E−09	Body
cg15818631	chr12		Open sea	0.26	0.27	2.58E−06	1.57E−10	
cg20721090	chr5	*MCC1*	Open sea	0.28	0.31	4.51E−10	5.24E−08	Body
cg24088279	chr16	*CHTF8*	Open sea	0.21	0.33	7.20E−06	4.86E−08	Body

**Fig. 5:**
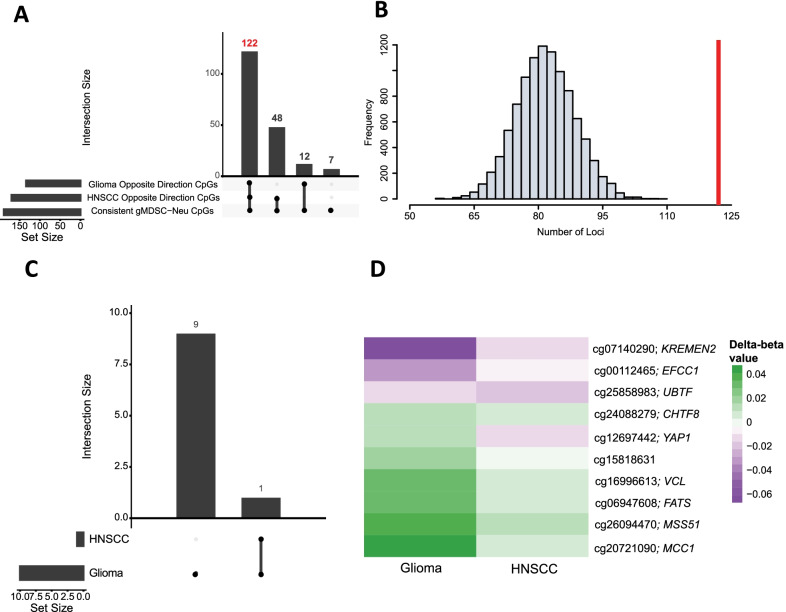
Assessing uniform change of whole blood DNAm in cancer studies for the 189 consistent loci and the core 10 loci. **A** Upset plot showing the overlap of the 189 DMRs with consistent gMDSC-Neu delta-betas in adult and cord blood samples and the respective CpG loci in the HNSCC and glioma studies that had case–control delta-betas in the opposite direction (termed “opposite direction CpGs”). **B** Histogram of the distribution under the null hypothesis of the number of loci with case–control delta-betas that are in the opposite direction of the respective gMDSC-Neu delta-beta in both the HNSCC and glioma study. Distribution was created by randomly drawing 189 CpG loci from the set of all loci with consistent gMDSC-Neu delta-betas and the counting the number of loci that have case–control delta-betas in the opposite direction of gMDSC-Neu delta-betas in both the glioma and HNSCC studies. The horizontal red line is at our observed value of 122 loci, far outside the range of the distribution. **C** Upset plot of CpGs that were significantly different between cases and cancer-free controls from testing the association between whole blood methylation beta-value and cancer status for each of the gMDSC core loci in two cancer studies. **D** Heatmap of the whole blood methylation delta-beta for cancer versus control in the glioma study population and the HNSCC study population. Each row represents one of the core ten loci. The color in the heatmap indicates the delta-beta value

Since numerous prior studies have demonstrated that gMDSC proportions are increased in the peripheral circulation of cancer patients, we reasoned that if alterations in the DNAm of the 189 CpGs represent gMDSCs, one might expect whole blood leukocyte DNAm of patients with cancers to change together, in a direction commensurate with the relatively small change in the prevalence of the MDSCs in whole blood. Importantly, we cannot interpret the meaning of the directionality of any change because we do not have leukocyte counts done at blood draw and numerous other cell types likely will change in concert with occurrence of the cancer. That is, since the relative numbers of leukocytes systematically change in cancer patients, the *proportional* change direction of any subtype (especially small subtypes) does not necessarily reflect the *numerical* change, although the direction would be hypothesized to be uniform. Hence, we compared the case–control delta-beta in the 189 consistent CpG loci signature from whole blood DNAm gMDSC in two cancer patient studies. In the HNSCC study, 170 CpG loci had a case–control delta-beta in the opposite direction of the corresponding gMDSC-Neutrophil delta-beta. In the glioma study, 134 CpG loci had a case–control delta-beta in the opposite direction of the corresponding gMDSC-Neutrophil delta-beta. Further, there was an overlap of 122 loci between these groups of CpG loci, meaning there were 122 loci that had a consistent case–control delta-beta in the two cancer studies that were opposite of the respective gMDSC-Neutrophil delta-beta found from adult and cord blood samples (Fig. 5A). To assess the chance of observing 122 loci with consistent delta-betas in these cancer studies that are also in the opposite direction of their respective gMDSC-Neutrophil delta-betas, we derived the distribution of the number of loci under the null hypothesis. This was done by: (1) creating a set of CpG loci with consistent gMDSC-Neutrophil delta-betas in adult and cord samples (*n* = 536,320 loci) (2) randomly sample 189 CpGs from this set (3) count the number of loci where the case–control delta-beta for both the HNSCC and glioma studies are the opposite direction of the respective gMDSC-Neutrophil delta-beta. This process was repeated 10,000 times and a histogram of the results are shown in Fig. 5B. The mean of this distribution is 82 and our observed value of 122 falls far outside the range. Of the core ten gMDSC signature loci, all ten CpGs were statistically significantly differentially methylated between cancer type and healthy controls in the larger glioma study, while for the less well-powered study of HNSCC patients, one loci was significantly different in cases compared with controls (Fig. 5C). When the difference in the estimate of DNA methylation (mean beta-value) between the control and cancer case was compared, the direction of the change was consistent in HNSCC and glioma for nine of ten of the loci (Fig. 5D).

## Discussion

In this study, we have directly compared the DNA methylation features of putative gMDSCs isolated from different sources and have identified the great heterogeneity of these cells at the epigenetic level, but also a core of common DNAm alterations. The isolation procedures used for the discovery samples captured a highly purified low-density population of cells with an immunophenotype and methylation signature consistent with neutrophil lineage. This result is in line with previous studies [10].

We have identified CpG loci that are differentially methylated between gMDSCs and neutrophils. Using two age-distinct populations of gMDSCs and neutrophils (adult and cord blood samples derived from diverse sources), we identified a set of 189 common CpG loci, with 10 CpG loci displayed consistent differential methylation between the cell subtypes. A number of the genes associated with the identified loci are known to have roles in immune responses, including some immune processes specific to neutrophils.

*VCL*, for example, codes for the scaffolding protein vinculin, which is involved in the maturation of integrin-based focal adhesions in a fashion previously termed ‘mechanosensitive’ [31]. Recent work has shown that this protein has a role in leukocyte trafficking, including a mechanosensitive role for vinculin in neutrophil adhesion and spreading [32]. Our work suggests, then, that vinculin may play an essential role in the genesis of particular features associated with gMDSCs. Several of the other core loci identified in this study have been shown to influence immune activity, through evidence of an association with the genesis of MDSCs. Deleting *FATS*, Fragile Site Tumor Suppressor, (one of the 10 common loci) in mice, has been shown to decrease the prevalence of gMDSCs in tumor tissues [33].

Expression of *YAP1*, or Yes-Associated Transcriptional Regulator, is associated with MDSC expansion [34]. Hyperactivated YAP1 signaling in tumors leads to MDSC recruitment [35], while blockage of *YAP1* leads to a decreased induction of MDSCs [34]. This suggests that *YAP1* expression is heightened in tumors and plays a role in recruiting and expanding immunosuppressive MDSCs. *YAP1* also seems to have a similar function as MDSCs in propagating tumor growth—it suppresses T-cell function and infiltration to the tumor microenvironment [36]. Though expression of *YAP1* by tumor cells propagates tumor growth, recent research has shown that there are binary pan-cancer classes where *YAP1* displays either pro- or anti-cancer activity depending on whether it is expressed or silenced in different types of cancer [37]. Hence, the role of *YAP1* in cancer seems to be complex, with our data suggesting that this gene has a direct role in gMDSC genesis.

*KREMEN2* (cg07140290), encoding Kringle-Containing TransmembraneProtein 2, is a protein implicated in cancer immunosurveillance in relation to WNT signaling [38]. Dysregulated WNT signaling culminates in cancer progression, malignant transformation, and resistance to treatment, undermining cancer immunosurveillance [38]. In the context of MDSCs, WNT displays anti-cancer effects as WNT/β-catenin signaling limits the tumor-promoting role of MDSCs, with downstream inhibition of β-catenin resulting in MDSC expansion and tumor infiltration [38, 39]. WNT/β-catenin within MDSCs themselves limits their ability to expand and infiltrate tumors. *KREMEN2* has potential involvement in these pathways because it hinders WNT activation [40].

Some of the identified loci are immunologically relevant, but their association with gMDSCs has not previously been recognized. Upstream Binding Transcription Factor (UBTF) plays a role in innate antiviral immunity as part of the pattern recognition receptors, where it partners with Interferon Gamma Inducible Protein 16 (IFI16) to restrict herpes simplex virus replication [41]. Interestingly, this process may also have a role in self-tolerance, as IFI16 forms oligomers spontaneously in Sjogren’s syndrome [42]. UBTF also contains a gamma activating sequence, suggesting it may operate as part of the interferon-gamma pathway [43]. The gene product of MCC Regulator of WNT Signaling Pathway (*MCC-1*) is similarly involved in the innate immune response [44], although its role in the genesis of gMDSCs is also unclear.

Finally, EF-Hand And Coiled-Coil Domain-Containing Protein 1 (*EFCC1*) has been studied in lung adenocarcinoma and shown, using immunohistochemical as well as RNA expression analysis, to be less prevalent in lung cancers and to predict poorer outcome [45]. Our data might suggest that some of the cells contributing to this assessment are infiltrating gMDSCs, with these having more methylated *EFCC1* and thus less protein and less RNA present. Regardless, our result, combined with the work of Xia et al. [45] does point to the potential utility of studies of both RNA and protein expression in tumors for study of MDSC infiltration. In terms of the other candidate loci (Table 2), cg15818631 does not have any associations with known genes, and *CHTF8* has no known role in the immune response.

After identifying these the common loci, we investigated their whole blood methylation status in cancer cases and healthy controls. The overlapping set of CpGs that represented gMDSCs in the cancer patients exhibited the same change in the direction of the methylation value compared with controls, suggesting that they reflect a co-methylation module, perhaps associated with coordinate differentiation. All of the core ten loci were significantly different between glioma cases and controls while 1 was significantly different in the smaller HNSCC study (possibly attributable to issues with power to detect small changes in the subtype prevalence); this could indicate that the pattern of altered methylation is partially attributable to an altered number of gMDSCs, which are known to be produced in cancer [6].

Strengths of this research include replication of our analyses in two distinct populations representative across the life-course—adult and cord blood. This work is particularly relevant to cancer, as most gMDSC samples came from cancer patients, and the results from discovery applied to cancer cases vs. cancer-free controls.

The use of heterogeneous populations might be a limitation, as some have suggested that low density cells in cord blood do not suppress lymphocyte mitogenesis, but rather this finding is an artifact of active macrophages that eliminate beads that stimulate T cell mitosis [46]. Regardless, the similar isolation procedures for both populations produced 190 CpGs with altered methylation that overlapped between these cell subtypes. Other limitations of this research include a relatively small sample size, although this allowed for the identification of ten loci with significantly altered methylation between gMDSCs and neutrophils in both adult and cord blood. While the scope of our research was to identify DNAm markers of gMDSCs, opportunities for future work include further investigation of these candidate loci. For example, lineage differentiation has long been known to be associated with microRNA expression [47] and further evaluation of the mechanistic importance of our findings should include detailed integration of these data with an assessment of altered microRNA. We’ve further investigated the whole blood DNAm of these loci in two different cancer types and found that nine of the ten loci were consistently different in cancer patients than controls. Together, our data indicate that gMDSCs have a core of distinct DNAm changes from neutrophils that inform future research on gMDSC differentiation and function.

## Supplementary Information

**Additional file 1: Table S1**. gMDSC deconvolution.

**Additional file 2: Table S2**. CpGs meeting criteria in adult blood samples.

**Additional file 3: Table S3**. Top 100 CpGs in adult samples.

**Additional file 4: Table S4**. Adult sample demograph.

**Additional file 5: Table S5**. CpGs meeting criteria in cord blood samples.

**Additional file 6: Table S6**. Top 100 CpGs in cord blood samples.

**Additional file 7: Table S7**. CpG characteristics.

**Additional file 8: Table S8**. Overlapping CpGs between adult and cord.

## Data Availability

The data generated in this study are available in the GEO database, upon acceptance of the paper in the journal.
